# Low-intensity pulsed ultrasound regulates osteoblast-osteoclast crosstalk via EphrinB2/EphB4 signaling for orthodontic alveolar bone remodeling

**DOI:** 10.3389/fbioe.2023.1192720

**Published:** 2023-06-23

**Authors:** Jie Zhou, Yanlin Zhu, Dongqing Ai, Mengjiao Zhou, Han Li, Yiru Fu, Jinlin Song

**Affiliations:** ^1^ College of Stomatology, Chongqing Medical University, Chongqing, China; ^2^ Chongqing Key Laboratory of Oral Diseases and Biomedical Sciences, Chongqing, China; ^3^ Chongqing Municipal Key Laboratory of Oral Biomedical Engineering of Higher Education, Chongqing, China

**Keywords:** low-intensity pulsed ultrasound, orthodontic tooth movement, alveolar bone remodeling, EphrinB2/EphB4 signaling, bone marrow mesenchymal stem cells, bone marrow monocytes

## Abstract

**Background:** The limited regenerative potential of periodontal tissue remains a challenge in orthodontic treatment, especially with respect to alveolar bone remodeling. The dynamic balance between the bone formation of osteoblasts and the bone resorption of osteoclasts controls bone homeostasis. The osteogenic effect of low-intensity pulsed ultrasound (LIPUS) is widely accepted, so LIPUS is expected to be a promising method for alveolar bone regeneration. Osteogenesis is regulated by the acoustic mechanical effect of LIPUS, while the cellular perception, transduction mode and response regulation mechanism of LIPUS stimuli are still unclear. This study aimed to explore the effects of LIPUS on osteogenesis by osteoblast-osteoclast crosstalk and the underlying regulation mechanism.

**Methods:** The effects of LIPUS on orthodontic tooth movement (OTM) and alveolar bone remodeling were investigated via rat model by histomorphological analysis. Mouse bone marrow mesenchymal stem cells (BMSCs) and bone marrow monocytes (BMMs) were purified and used as BMSC-derived osteoblasts and BMM-derived osteoclasts, respectively. The osteoblast-osteoclast co-culture system was used to evaluate the effect of LIPUS on cell differentiation and intercellular crosstalk by Alkaline phosphatase (ALP), Alizarin Red S (ARS), tartrate-resistant acid phosphatase (TRAP) staining, real-time quantitative PCR, western blotting and immunofluorescence.

**Results:** LIPUS was found to improve OTM and alveolar bone remodeling *in vivo*, promote differentiation and EphB4 expression in BMSC-derived osteoblasts *in vitro*, particularly when cells were directly co-cultured with BMM-derived osteoclasts. LIPUS enhanced EphrinB2/EphB4 interaction between osteoblasts and osteoclasts in alveolar bone, activated the EphB4 receptor on osteoblasts membrane, transduced LIPUS-related mechanical signals to the intracellular cytoskeleton, and gave rise to the nuclear translocation of YAP in Hippo signaling pathway, thus regulating cell migration and osteogenic differentiation.

**Conclusions:** This study shows that LIPUS modulates bone homeostasis by osteoblast-osteoclast crosstalk via EphrinB2/EphB4 signaling, which benefits the balance between OTM and alveolar bone remodeling.

## 1 Introduction

The insufficient regeneration of periodontal tissue remains a vital problem for orthodontic treatment, with the mismatch between the remodeling of alveolar bone and the rate of orthodontic tooth movement (OTM) limiting clinical treatment at present. Clinically, the slow speed of OTM leads to a long orthodontic treatment course, and insufficient alveolar bone formation after OTM results in periodontal support tissue defects, dehiscence, fenestration and other iatrogenic injuries (Danz et al., 2016; Huang et al., 2016; Antoun et al., 2017). Therefore, improving the quality and efficiency of orthodontic alveolar bone remodeling in order to achieve safe and efficient orthodontic treatment is a current research focus. To date, many studies on adjuvant therapies for orthodontic alveolar bone remodeling, including surgery, drugs, physiotherapy, biomaterials, *etc.*, have been published (de Almeida et al., 2016; Gao et al., 2021; Lu et al., 2022). However, in the process of orthodontic alveolar bone remodeling, promoting bone formation to ensure periodontal health and promoting bone absorption to accelerate tooth movement are like two sides of a coin. Existing research often ignores the balance between these two goals.

The therapeutic effect of Low-intensity Pulsed Ultrasound (LIPUS) on tissue regeneration has been widely demonstrated in recent years. Animal and clinical trials have demonstrated that LIPUS can improve the tissue repair and regeneration potential, shorten the healing time in bone and soft tissue injuries, and is a non-invasive, safe and effective treatment for delayed fracture healing and bone non-union (Martinez de Albornoz et al., 2011; Lai et al., 2021; Xia et al., 2022). Studies have also shown that LIPUS can accelerate OTM (Kaur and El-Bialy, 2020), reduce root resorption during orthodontic treatment (El-Bialy et al., 2020), and promote proliferation and osteogenesis of periodontal ligament cells (PDLCs) (Alazzawi et al., 2018). Our previous studies have also confirmed that LIPUS can effectively improve the regeneration of bone defects in periodontitis (Wang et al., 2018; Li et al., 2020; Ying et al., 2020). Thus, it is speculated that LIPUS may be a suitable approach to promote the remodeling of the alveolar bone.

In bone remodeling, maintaining the balance between bone absorption and formation is essential in order to preserve the morphological structure of the bone and its normal function. Recent studies have suggested that the bidirectional signal between EphB4 receptors on osteoblasts and EphrinB2 ligands on osteoclasts plays a role in modulating bone homeostasis (Kim et al., 2020). Activating EphrinB2/EphB4 signaling can promote osteoblast differentiation while inhibiting osteoclast differentiation (Li et al., 2015; Sen et al., 2015).

Therefore, this study aimed to 1) explore the effects of LIPUS on alveolar bone remodeling during OTM, 2) evaluate the effects of LIPUS on the differentiation of bone mesenchymal stem cells (BMSCs)-derived osteoblasts in an osteoblast-osteoclast co-culture system, and 3) identify the role of EphrinB2/EphB4 signaling in this process.

## 2 Results

### 2.1 LIPUS increases the rate of OTM and compensatory bone formation in rats

**FIGURE 1:**
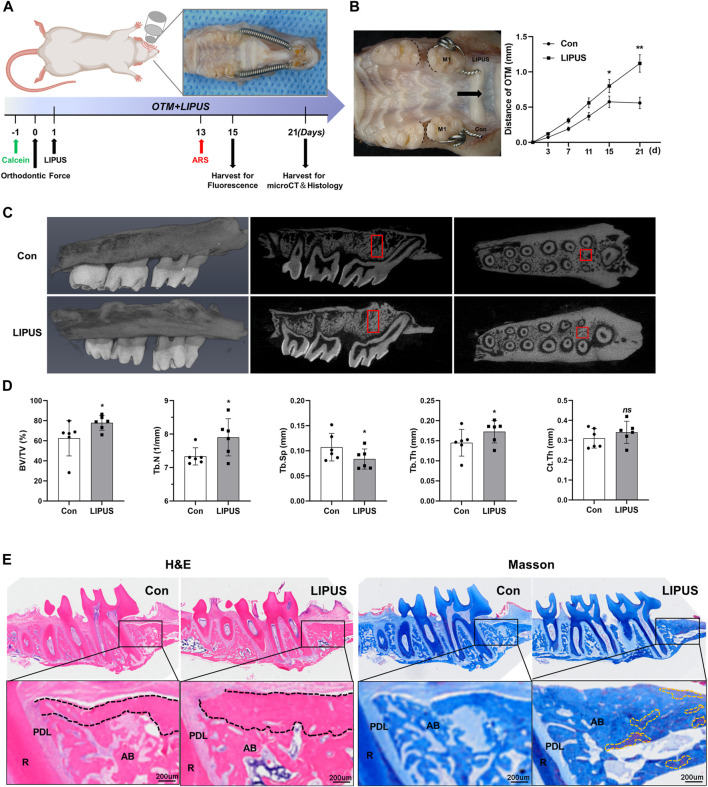
Low-intensity pulsed ultrasound (LIPUS) accelerates orthodontic tooth movement (OTM) and modulates alveolar bone remodeling. **(A)** Scheme of LIPUS treatment in OTM experiment. **(B)** Effect of LIPUS on the rate of OTM (left); the distance was increased by LIPUS on days 15 and 21 (right), n = 3. **(C)** Representative 3D reconstructed images (left), sagittal direction images (mid) and transverse direction images (right) of the maxillary molars examined by micro-computed tomography. **(D)** Quantification of bone histomorphometry including BV/TV, Tb.N, Tb.Th, Tb. Sp and Ct. Th, n = 6. **(E)** Hematoxylin-Eosin (HE) and Masson staining images of alveolar bone on the pressure side on day 21 (scale bar = 200 μm). R: root; PDL: periodontal ligament; AB: alveolar bone. (Black dashed lines represent cortical bone; yellow dashed lines represent mature new bone). Data are presented as the mean ± SEM. *p < 0.05, **p < 0.01 and ns = no significance represent the statistical significance of the difference between the indicated groups.

To investigate the effects of LIPUS irradiation on the rate of OTM and alveolar bone remodeling, OTM models on the bilateral maxillary first molars of rats were constructed; a schematic of the study flow is presented in Figure 1A. LIPUS accelerated OTM from the third day, and there were statistically significant differences in the tooth movement distance on the 15th and 21st days (Figure 1B). Given that the alveolar bone between the mesial and distal roots of the first molar includes both tension and pressure areas during OTM, the changes in the alveolar bone volume and microstructure of the region of interest (ROI) were evaluated using micro-computed tomography (micro-CT). Representative reconstructed images and sectional images in different directions are shown in Figure 1C. The LIPUS group showed more movement compared to the control group, although it was not entirely horizontal. It can be seen that on the 21st day, there was a smaller OTM distance and fewer bone trabeculae in the alveolar cancellous bone, with a clear porous structure on the control side. The quantitative results indicated that LIPUS treatment increased the bone volume/total volume (BV/TV), trabecular number (Tb.N) and trabecular thickness (Tb.th), and decreased the trabecular separation (Tb.Sp) compared with the control group during OTM (Figure 1D). Next, the histological changes in the alveolar bone around the first molar after 21 days of OTM under LIPUS treatment were observed using hematoxylin-eosin (HE) and Masson staining. Similar to the micro-CT results, the LIPUS side had more newly deposited bone at the edge of the trabecula in the alveolar cancellous bone and the bone trabeculae were thick and closely arranged. Higher new bone formation in cortical and cancellous bones of LIPUS side, indicating promising bone formation potential. In addition, the proximal buccal cortex of the orthodontic teeth treated by LIPUS was significantly thickened and suggesting that LIPUS may promote compensatory bone formation during OTM (Figure 1E). Micro-CT analysis showed that LIPUS stimulation slightly increased the cortical bone thickness on the pressure side, although there was no statistical difference in Ct.Th, which may be due to insufficient cortical mineralization during the shorter experimental period (Figure 1D).

**FIGURE 2:**
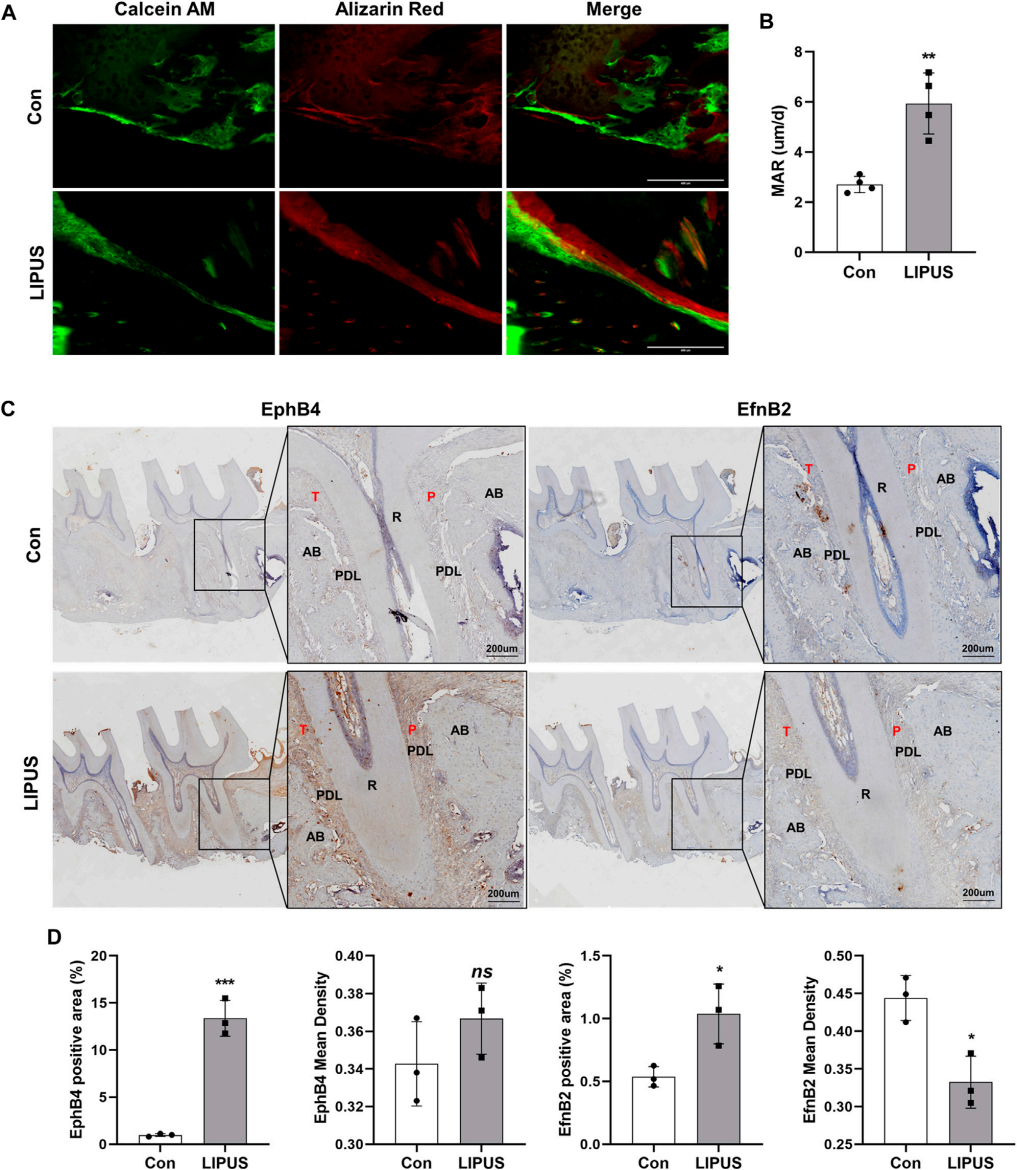
LIPUS improves compensatory bone formation in OTM via EphrinB2/EphB4 signaling. **(A)** Representative images of alveolar bone cortex on the pressure side labelled with Calcein and alizarin red S (ARS) (scale bar = 400 μm). **(B)** Quantification of mineral apposition rate (MAR) of the control and LIPUS sides, n = 4. **(C)** Representative immunohistochemical staining images of EphB4 and EphrinB2 at ×200 (left) and ×400 (right) magnification (scale bar = 200 µm). **(D)** The quantitative analysis of EphB4 and EphrinB2 expression by the positive area and average optical density of the alveolar bone, n = 3. Data are presented as the mean ± SEM. *p < 0.05, **p < 0.01 and ***p < 0.001 represent the statistical difference between the indicated groups.

To further explore the effect of LIPUS on bone homeostasis during OTM, calcein and alizarin red S (ARS) fluorescent labels were used to analyse the regions of bone formation. As shown in Figure 2A, the fluorescence signal of the LIPUS side was significantly enhanced, and the distance between the two labelled edges was increased. The bone mineral apposition rate (MAR) of the LIPUS side was significantly greater than that of the control (Figure 2B). These results confirm that LIPUS promotes changes in compensatory osteogenesis in the bone cortex on the pressure side during OTM in rats. To test the hypothesis that EphrinB2/EphB4 signaling may be involved in LIPUS mechanical signal sensing and transduction, the expression patterns of EphB4 and EphrinB2 in the periodontal ligament (PDL) and alveolar bone were examined by Immunohistochemistry (IHC). The results showed that EphB4 was expressed in both the PDL and alveolar bone, while EphrinB2 was mainly expressed in the alveolar bone and was rarely found in the PDL (Figure 2C). Quantitative analysis showed that the positive areas of EphB4 and EphrinB2 were more widely distributed in the LIPUS group, although the average optical density of EphrinB2 was decreased compared with the control side, suggesting the effect of LIPUS on alveolar bone remodeling may be achieved by increasing the binding between EphB4-EphrinB2 (Figure 2D). These findings suggest that LIPUS affects the expression of the EphrinB2/EphB4 signal during OTM, and thus, may influence the homeostasis balance of the orthodontic alveolar bone, resulting in compensatory bone cortex formation through osteoblast-osteoclast crosstalk.

### 2.2 LIPUS promotes osteogenesis of BMSC-derived osteoblasts in co-culture system with bone marrow monocytes (BMMs)-derived osteoclasts

**FIGURE 3:**
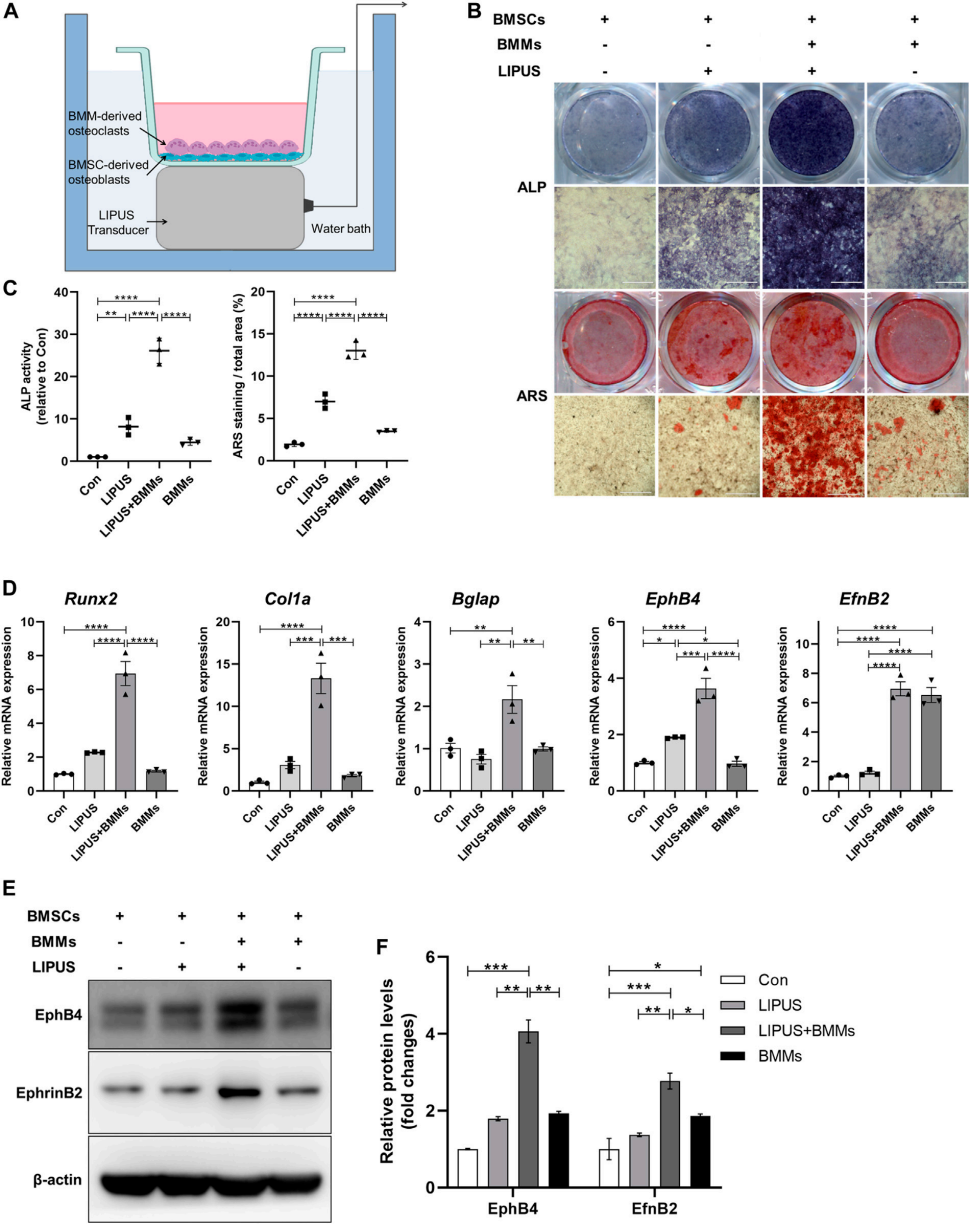
LIPUS promotes the differentiation of bone marrow mesenchymal stem cells (BMSCs)-derived osteoblasts in co-culture system. **(A)** Schematic of the LIPUS treatment on the BMSC-derived osteoblasts and bone marrow monocytes (BMMs)-derived osteoclasts in the co-culture system. **(B)** Representative images of ALP and ARS staining of each group on days 7 and 21 after osteogenic induction, respectively. **(C)** Alkaline Phosphatase (ALP) activity measurement of cell lysates after osteoinduction for 7 days (left) and quantification of mineralized nodules (right). **(D)** Real time-quantitative PCR (RT-qPCR) analysis of the relative expression of Runx2, Col1a, Bglap, EphB4 and EphrinB2. **(E, F)** Relative protein levels of EphB4 and EphrinB2 were determined by Western blotting in BMSCs co-cultured with BMMs in the absence or presence of LIPUS. Data are presented as the mean ± SEM, n = 3. *p < 0.05, **p < 0.01, ***p < 0.001 and ****p < 0.0001 represent the statistical difference between the indicated groups.

BMSC-derived osteoblasts in mono-culture or in co-culture system with BMM-derived osteoclasts were studied using the methods shown in Figure 3A. First, we attempted to determine the effect of LIPUS on the differentiation of osteoblasts. Alkaline Phosphatase (ALP) and ARS staining revealed that, compared with the control, BMSC-derived osteoblasts that were exposed to LIPUS treatment had increased ALP activity and mineralized nodule formation, which is consistent with previous research reports. Interestingly, BMSC-derived osteoblasts in the direct co-culture system with BMMs had significantly increased ALP activity and formation of mineralized nodules following LIPUS treatment, suggesting that BMM-derived osteoclasts may work in synergy with LIPUS to promote the osteogenesis of BMSC-derived osteoblasts. To explore whether BMMs themselves affected the osteogenic potential of BMSC-derived osteoblasts, ALP activity and calcium salt deposition were evaluated in the osteoblast-osteoclast co-culture system without LIPUS treatment. The results revealed no significant changes compared with BMSC-derived osteoblasts in mono-culture (Figures 3B, C). The osteogenic phenotype of BMSC-derived osteoblasts in each group was determined by real time-quantitative PCR (RT-qPCR). The results showed that the mRNA expression levels of the osteogenic specific transcription factor Runx2 and the early osteogenic differentiation marker gene Col1a in osteoblasts were slightly increased by LIPUS treatment, and significantly increased by the combination of osteoclast co-culture and LIPUS stimulation. Meanwhile, there was no significant enhancement in osteogenic gene expression in the osteoblast-osteoclast co-culture system without LIPUS treatment. The mRNA expression of the cell membrane receptor EphB4 was upregulated by LIPUS stimulation, and its expression was significantly increased in the osteoclast co-culture system with LIPUS treatment, while the expression of the cell surface ligand EphrinB2 did not change significantly under the action of LIPUS (Figure 3D). The protein expression levels of EphB4 and EphrinB2 in each group were consistent with the transcriptional levels (Figures 3E, F).

### 2.3 Effect of LIPUS on differentiation of BMM-derived osteoclasts in the co-culture system

**FIGURE 4:**
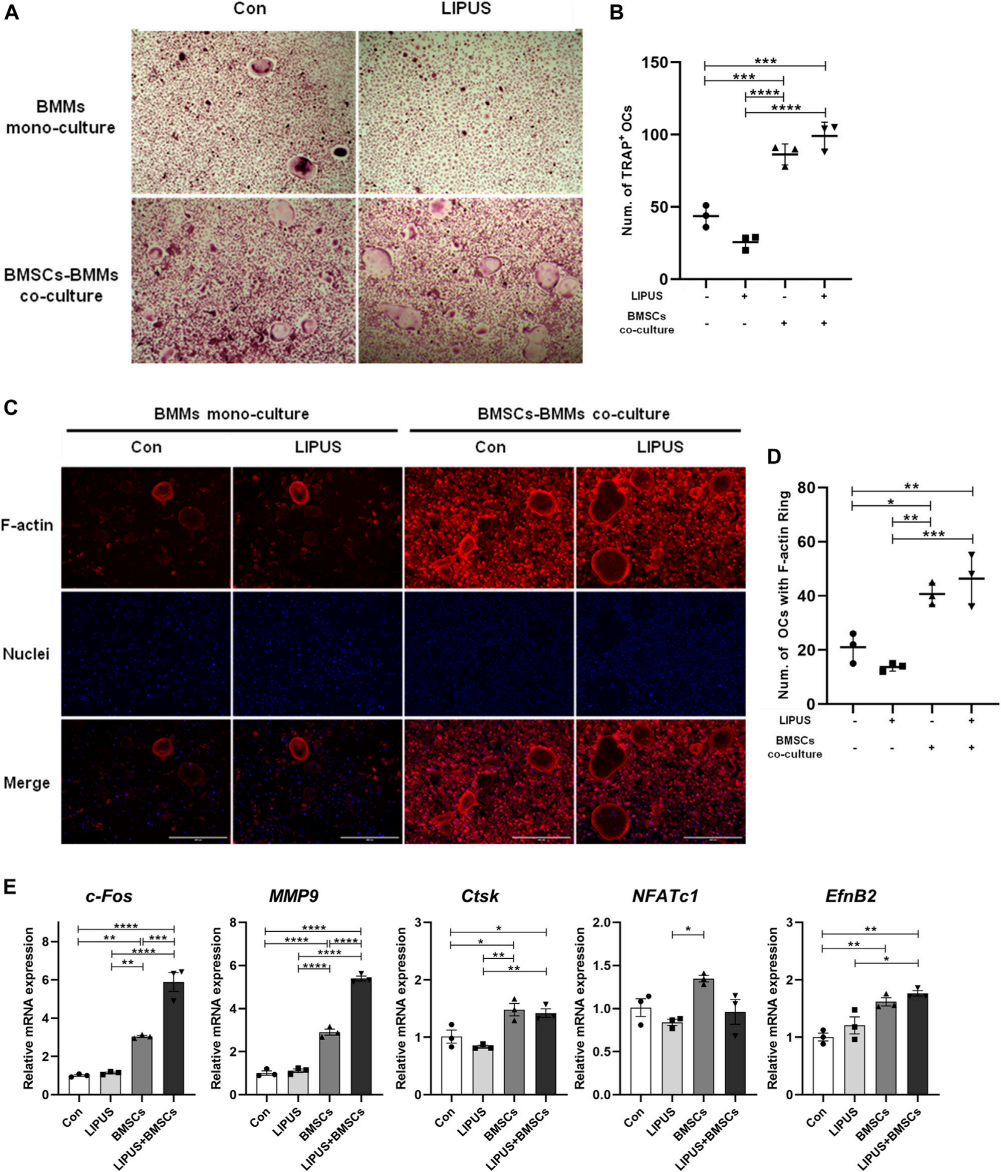
Effect of LIPUS on differentiation of BMM-derived osteoclasts in the co-culture system. **(A–C)** TRAP staining and F-actin ring staining of BMM-derived osteoclasts at day seven of osteoclastogenesis (scale bar = 400 µm). **(B–D)** The count of TRAP-positive mononuclear/multinuclear osteoclasts and the number of osteoclasts with F-actin rings were quantified in each well. **(E)** RT-qPCR analysis of the relative expression of osteoclastogenesis-related genes including c-fos, Nfatc1, MMP9, Ctsk and EphrinB2. Data are presented as the mean ± SEM, n = 3. *p < 0.05, **p < 0.01, ***p < 0.001 and ****p < 0.0001 represent the statistical difference between the indicated groups.

Since EphrinB2/EphB4 signals have bidirectional regulatory effects on the osteoblasts and osteoclasts, we sought to determine the effect of LIPUS on osteoclast differentiation in the co-culture system. LIPUS was applied to the BMMs in mono-culture and in co-culture system with BMSC-derived osteoblasts. Then, tartrate-resistant acid phosphatase (TRAP) staining and F-actin ring fluorescence was used to evaluate the osteoclast differentiation of BMMs. The results showed that LIPUS had no significant effects on the number and size of osteoclasts in the mono-culture BMMs. Regardless of the presence of LIPUS stimulation, the number and size of osteoclasts were increased (Figures 4A, B) and the F-actin ring of the osteoclast skeleton was significantly visible (Figures 4C, D) in the BMM-derived osteoclasts in the co-culture system with osteoblasts. This suggests that the formation and maturation of osteoclasts were upregulated by the combination of BMSC-derived osteoblasts. RT-qPCR results showed that LIPUS had no significant effects on osteoclast differentiation genes in mono-culture BMMs, but slightly increased the c-fos and MMP9 mRNA expression levels of osteoclasts in the osteoblast cell co-culture system. This suggests that LIPUS promotes the differentiation and maturation of osteoclasts in a co-culture system (Figure 4E). At the same time, EphrinB2 mRNA, mainly expressed on the membrane of osteoclasts, was significantly increased in BMM-derived osteoclasts in the osteoblast cell co-culture system compared with the mono-culture BMMs; however, its expression was not affected by LIPUS.

### 2.4 LIPUS enhances BMSC-derived osteoblasts differentiation in combination with EphrinB2-Fc simulated forward signaling

**FIGURE 5:**
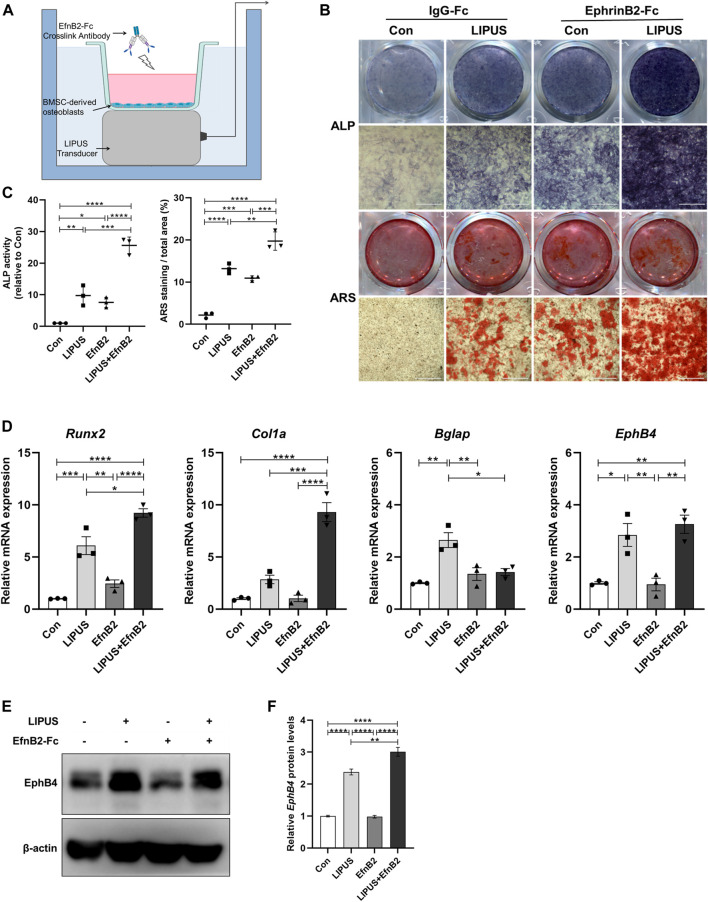
LIPUS enhances osteogenic differentiation in combination with EphrinB2-Fc simulated forward signal. **(A)** Schematic of LIPUS and EphrinB2-Fc preclustered with Anti-His-tag treatment on BMSC-derived osteoblass. **(B)** Representative images of ALP and ARS staining of each group on days 7 and 21 after osteogenic induction, respectively. **(C)** ALP activity measurement of cell lysates after osteoinduction for 7 days (left) and quantification of mineralized nodules (right). **(D)** RT-qPCR analysis of the relative expression of osteogenesis-related genes including Runx2, Col1a, Bglap and EphB4. **(E, F)** Relative protein levels of EphB4 were determined by Western blotting in BMSCs incubated with EphrinB2-Fc in the absence or presence of LIPUS. Data are presented as the mean ± SEM, n = 3. *p < 0.05, **p < 0.01, ***p < 0.001 and ****p < 0.0001 represent the statistical difference between the indicated groups.

To mimic the specific ligand to EphB4 on the osteoclast surface, recombinant mouse EphrinB2-Fc chimera protein was utilized to study whether EphrinB2 is involved in the regulatory effect of LIPUS on BMSCs osteogenesis. The methods utilised in this part of the study are shown in Figure 5A. ALP and ARS staining were used to evaluate the osteogenic ability of each group, and the results indicated that the osteogenic effect was strongest for the combination of LIPUS and EphrinB2-Fc, followed by the single-stimulus conditions of LIPUS or EphrinB2-Fc (Figures 5B, C). RT-qPCR and Western blotting were used to evaluate the expression changes in osteogenesis-related marker genes and EphB4. Compared with the control group, LIPUS increased the mRNA expression levels of Runx2, Bglap and EphB4 in BMSC-derived osteoblasts, but there was no significant difference in the osteogenesis genes expression by EphrinB2-Fc single treatment. With the combined stimulation of LIPUS and EphrinB2-Fc, osteoblastic differentiation was increased remarkably (Figure 5D). Moreover, the Western blotting results confirmed that EphB4 protein expression was increased with LIPUS stimulation, and EphrinB2-Fc enhanced this upregulation (Figures 5E, F).

### 2.5 EhpB4 depletion impairs osteogenesis modulated by LIPUS and EphrinB2-Fc

**FIGURE 6:**
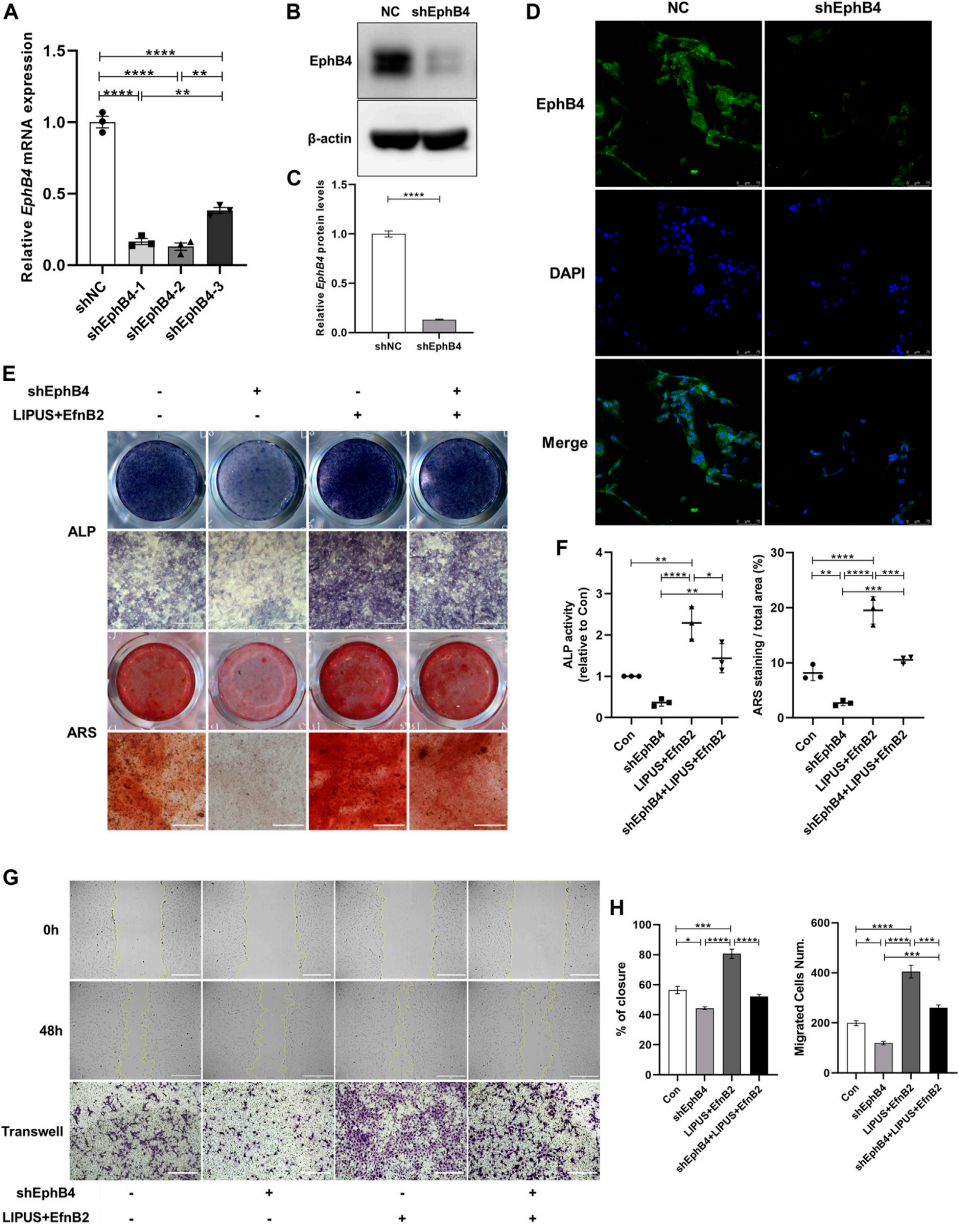
EhpB4 depletion impairs the osteogenesis induced by LIPUS and EphrinB2-Fc. **(A)** RT-qPCR analysis of the relative expression of EphB4 in BMSCs transfected with shEphB4 plasmid. **(B–C)** Relative protein levels of EphB4 were determined by Western blotting. **(D)** Immunofluorescence staining of EphB4 after transfection with shEphB4 in BMSCs (scale bars = 75 µm). **(E)** Representative images of ALP and ARS staining of each group on days 7 and 21 after osteogenic induction, respectively. **(F)** ALP activity measurement of cell lysates after induction for 7 days (left) and quantification of the mineralized nodules (right) in each group. **(G)** The images depict the migratory ability of different groups as measured by wound healing from 0 h (upper) to 48 h (mid) and Transwell migration assays (lower) in BMSC-derived osteoblasts (scale bar = 400 μm). **(H)** Quantitative analysis of relative wound closure and migrated cell number was performed using ImageJ software. Data are presented as the mean ± SEM, n = 3. *p < 0.05, **p < 0.01, ***p < 0.001 and ****p < 0.0001 represent the statistical difference between the indicated groups.

To explore the role of EphB4 as an EphrinB2 receptor in LIPUS signal perception and BMSCs osteogenesis regulation, we tested the biological function of EphB4 by knocking it down in BMSCs. ShEphB4-2 was selected due to the higher efficiency of interference through qPCR (Figure 6A). The Western blot and immunofluorescence results confirmed that shEphB4 effectively reduced the protein level of EphB4 (Figures 6B–D). ALP and ARS staining results indicated that knockdown of EphB4 by shRNA led to a decrease in ALP activity and mineralization in BMSCs. However, with the combination of EphrinB2-Fc, LIPUS showed great osteogenic potential, while this effect was remarkably attenuated by shEphB4 (Figures 6E, F). This suggests that the forward signal of EphrinB2-EphB4 may be involved in the effect of LIPUS on BMSCs osteogenesis, but it may not be the only factor contributing to this effect. Evaluation of wound healing and the transwell test revealed that the synergistic effect of LIPUS and EphrinB2-Fc significantly improved the migration ability of BMSCs, while EhpB4 depletion impaired the enhanced migration ability of BMSCs (Figures 6G, H). This suggests that LIPUS may affect the migration of BMSCs through EphB4 signaling, thereby affecting the osteogenesis of BMSCs.

### 2.6 YAP Signaling participates in the response and transduction of the EphrinB2/EphB4 signal in LIPUS-induced osteogenesis

**FIGURE 7:**
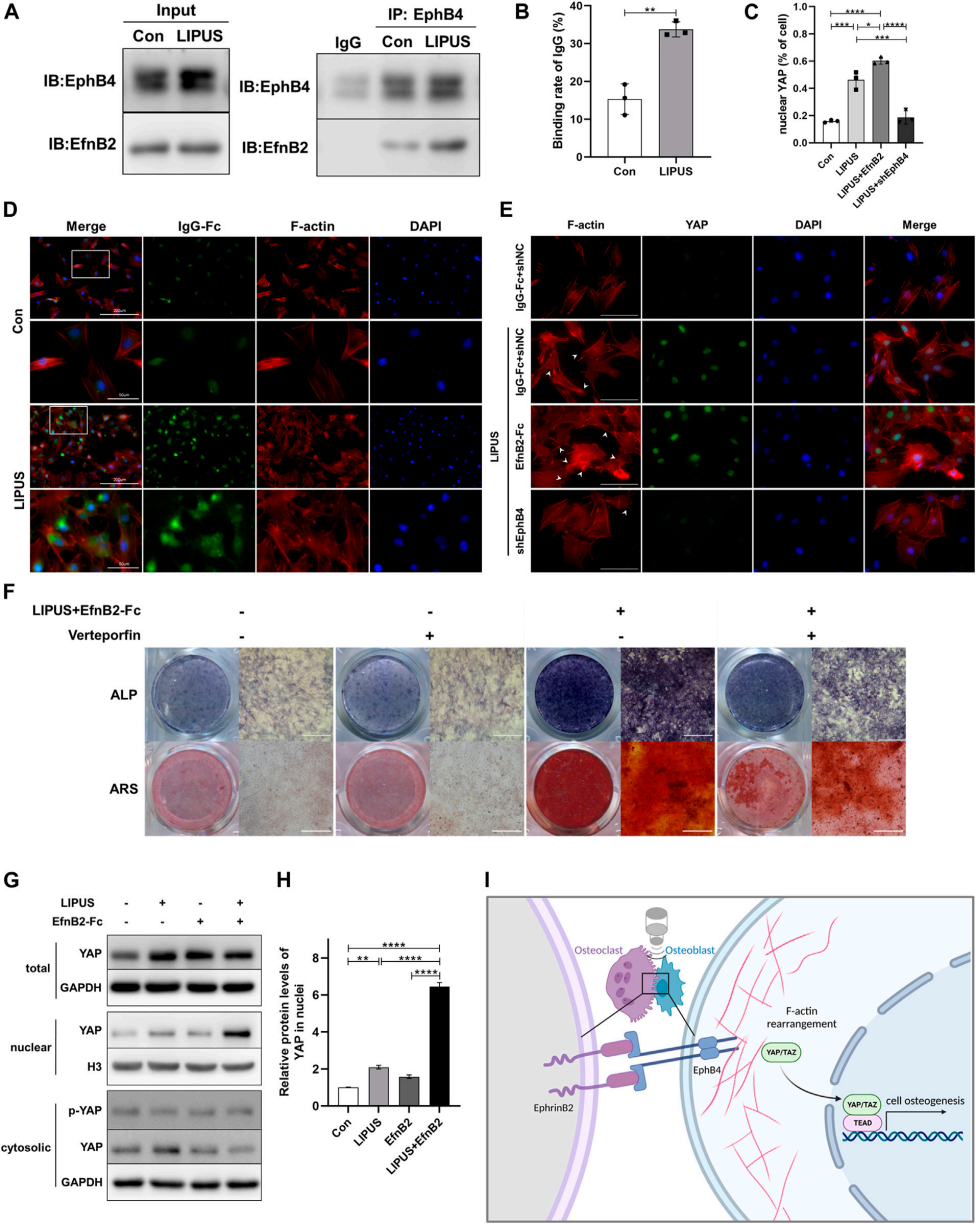
YAP participates in the signal transduction of EphrinB2/EphB4 in LIPUS-induced osteogenesis through actin filaments. **(A)** Co-IP experiments showed the interaction between EphB4 and EphrinB2 in the co-culture system as immunoprecipitation with anti-EphB4 antibody to enrich the protein complex. **(B)** The binding rate of IgG on BMSC-derived osteoblasts, defined as the rate of osteoblasts with attached EphrinB2-Fc among the total osteoblasts, n = 3. **(C)** Quantification of the proportion of YAP localized in nucleus in BMSC-derived osteoblasts by immunofluorescence using ImageJ, n = 3. **(D)** Immunofluorescent staining of crosslink antibody (green) to detect EphrinB2-Fc and F-Actin to show cytoskeleton (red). Lower images (scale bar = 50 μm) are high magnification of the white squares in the corresponding upper images (scale bar = 200 μm). **(E)** YAP nuclear translocation and stress fibres of the cytoskeleton were determined by immunofluorescence (scale bar = 100 µm). Green: YAP staining, Red: F-actin, Blue: nucleus (DAPI), White arrow: filopodia and lamellipodia. **(F)** Representative images of ALP staining and ARS staining for mineralized nodules on days 7 and 21 after osteogenic induction, respectively. **(G, H)** The nuclear-cytoplasmic fraction was separated from BMSC-derived osteoblasts treated with LIPUS or EphrinB2-Fc. Immunoblotting assay showed that LIPUS in combination with EphrinB2-Fc treatment significantly increased YAP nuclear localization and slightly decreased phosphor-YAP in cytoplasm, n = 3. **(I)** Schematic diagram (created with biorender.com) shows that EphrinB2/EphB4 is involved in regulating migration and osteogenesis of BMSC-derived osteoblasts in a co-culture system under mechanical stimulation with LIPUS. Mechanistically, EphrinB2/EphB4 functions as mechanotransducer in response to the piezoelectric and micro-vibration effect of LIPUS and regulates YAP nuclear translocation through F-actin filament rearrangement. Data are presented as the mean ± SEM, n = 3. *p < 0.05, **p < 0.01, ***p < 0.001 and ****p < 0.0001 represent the statistical difference between the indicated groups.

Having revealed the LIPUS promotes the migration and osteogenesis of BMSCs by increasing the expression of EphB4 on the membrane of BMSC-derived osteoblasts, especially under the co stimulation of osteoclasts or EphrinB2-Fc, we further sought to reveal the underlying molecular mechanism. To determine the effect of LIPUS on the interaction between EphrinB2 of osteoclasts and EphB4 of osteoblasts, the co-culture system was treated with LIPUS stimulation and osteogenic medium for 7 days. The immunoprecipitation results revealed that LIPUS significantly increased the binding between endogenous EphrinB2 and the EphB4 protein (Figure 7A). The immunofluorescence of His-Tag-antibody cross-linked with EphrinB2-Fc chimera protein represents the amount of EphrinB2-Fc binding to the EphB4 receptor on the osteoblast membrane; the results indicated that LIPUS facilitated the attachment of EphrinB2-Fc to EphB4 (Figures 7B, D). Previous studies have reported that the cytoskeleton functions as a converter for MSC differentiation by responding to EphB4 then affecting YAP nuclear translocation. To study the structure of the stress fibre response to LIPUS stimulation, F-actin was stained and the fluorescence intensity of YAP in nuclei was quantified. The results indicated that, in the control group, F-actin was arranged along the long axis of the cell in the form of filaments. LIPUS and EphrinB2-Fc promoted the gathering of F-actin filaments around the nucleus and the formation of filopodia and lamellipodia on the cell membrane, while the depletion of EphB4 mitigated the changes in F-actin and YAP caused by LIPUS (Figures 7C, E). These results suggest that EphB4, as a membrane receptor, exhibits enhanced binding with EphrinB2 following LIPUS treatment, and then affects YAP nuclear localization through cytoskeleton rearrangement and downstream target genes. Western blotting for nucleocytoplasmic separation showed that LIPUS slightly increased the overall abundance of YAP in BMSC-derived osteoblasts, significantly promoted the nuclear localization of YAP, and slightly reduced its phosphorylation in the cytoplasm with the coordination of EphrinB2-Fc (Figures 7G, H). Finally, to determine the biological role of YAP in the transduction of the LIPUS- EphB4- EphrinB2- F-actin cascade signal, Verteporfin was added to the cells to block the role of YAP. The results of ALP and ARS staining indicated that YAP blocking reduced the activity of ALP and the level of calcium salt deposition, supporting the speculation that the positive regulatory effect of EphB4 on osteogenesis may be related to YAP signaling (Figure 7F).

## 3 Discussion

Orthodontic alveolar bone remodeling is essentially a bone homeostasis process under the influence of mechanical stress (Dieterle et al., 2021; Wang et al., 2022). When orthodontic force is applied to teeth, cells such as BMSCs and PDLCs in the periodontal ligament tissue on the tension side can differentiate to osteoblasts and form new alveolar bone. In the proper alveolar bone on the pressure side, the osteoclasts are active and cause bone absorption, resulting in tooth movement (Li Y. et al., 2021; Brockhaus et al., 2021). While bone remodeling of the alveolar bone cortex on the pressure side, i.e., compensatory osteogenesis, is of great significance to maintaining the thickness of the alveolar bone after tooth movement, the remodeling is often insufficient and can cause dehiscence and fenestration (Sheng et al., 2020; Dominiak et al., 2021; Sun et al., 2022). Therefore, ideal orthodontic treatment requires rapid completion of tooth movement without irreversible damage to the root and periodontal tissue during the process. Given that LIPUS can improve tissue regeneration and repair (Martinez de Albornoz et al., 2011; Lai et al., 2021; Xia et al., 2022), the application of LIPUS in dentistry has also been increasingly explored (Miura et al., 2014; Toy et al., 2014; Kang et al., 2016). Recently, it has been reported that LIPUS can accelerate OTM by upregulating osteoclasts (Xue et al., 2013; Feres et al., 2016). Our study proved that LIPUS can significantly increase the OTM as expect. Micro-CT and histomorphology revealed increased osteogenesis on the tension side, suggesting that LIPUS promotes the osteogenic differentiation of PDLCs or BMSCs; this is consistent with other published studies (Shirakata et al., 2020; Shobara et al., 2021). Of most interest was the observation that the lateral bone cortex on the pressure side was thickened and compensatory osteogenesis was increased; this is of significance for maintaining the thickness of the pressure side alveolar bone in orthodontic treatment, and is consistent with a previous report (Arai et al., 2020). It is hypothesized that this compensatory bone formation may be attributed to the effect of LIPUS on the signal transduction of cells communication.

The structure of bone tissue must be maintained through a strictly regulated bone remodeling process. Disrupting the complex interactions between these different cell systems could lead to bone diseases (Plotkin and Bellido, 2016). The interaction between cells during orthodontic alveolar bone remodeling is closely related to the activity of osteoblasts or osteoclasts and the microenvironment. BMSCs and osteoblasts, which dominate bone formation, are affected by osteoclasts, macrophages and vascular endothelial cells *in vivo*, thereby affecting bone formation (Hathaway-Schrader and Novince, 2000; Wang et al., 2021). Thus, it is difficult to comprehensively understand the mechanism underlying the effect of LIPUS on alveolar bone remodeling using just a single-cell model. Accordingly, co-culture system is a more reasonable cell culture model which can be used to simulate the microenvironment of cell co-growth, providing a good means to evaluate the biological behaviour of cells (Borciani et al., 2020; Hong et al., 2022). Based on the *in vivo* results which indicated that LIPUS promotes osteogenesis and osteoclast differentiation, it was hypothesized that LIPUS may regulate osteoclast and osteoblast formation at the same time in the co-culture system. ALP and ARS staining showed that LIPUS enhanced the differentiation of BMSC-derived osteoblasts, which is consistent with existing reports (Xia et al., 2022). The osteogenic induction effect of LIPUS was significant in the direct co-culture system but not in the indirect co-culture system. This may be because LIPUS enhances osteogenic differentiation through direct contact signal transduction in the osteoblast-osteoclast co-culture system rather than via paracrine cell communication. On the other hand, the results indicated that LIPUS had no significant effect on osteoclast differentiation in mono-culture BMMs, but induced osteoclast differentiation in the osteoblast-osteoclast co-culture system. This suggests that the differentiation and maturation of osteoclasts require the interaction of osteoblasts. However, the result is in contradiction with the finding that EphB4 could inhibits osteoclast differentiation as a reverse signal (Ge et al., 2020). Thus, we speculate that this effect may be through other signaling pathways; for example, the RANKL/OPG pathway of osteoblasts may be increased by LIPUS.

LIPUS produces a mechanical signal that is characterised by piezoelectric micro-vibration; this signal must be sensed and converted into biochemical signals to generate biological effects. This occurs through different mechanoreceptors, including integrin, stress-activated ion channels and membrane receptors, which act as mediators between the cytoskeleton and the extracellular matrix (Zhang et al., 2022). Recent reports suggest that EphB4-EphrinB2, a member of the tyrosine receptor family, plays a direct role in bone development, regeneration and alveolar bone remodeling, and is regulated by stress stimulation (Baek et al., 2018; Li M. et al., 2021). The results showed that EphrinB2 was similar to the function of osteoclasts in the osteoblast-osteoclast co-culture system; this is consistent with a previous report (Diercke et al., 2011; Zhu et al., 2017). On the other hand, regardless of the presence or absence of EphrinB2-Fc, EphB4 knockdown impaired the osteogenic effect of LIPUS, and LIPUS affected the migration of BMSCs by EphB4. Together, these findings suggest that LIPUS not only increases BMSCs migration but also promotes osteogenesis through EphrinB2/EphB4 positive signaling, thereby enhancing remodeling of the alveolar bone cortex on the pressure side of orthodontic teeth and improving the safety of orthodontic alveolar bone remodeling.

Further, we explored how LIPUS affected the binding of EphrinB2 and EphB4, and how its downstream signals affected osteogenesis. The Co-IP and immunofluorescence indicated that the mechanical effect of LIPUS may increase the migration ability of BMSCs and the crosstalk of EphB4 receptor to EphrinB2 ligand. Studies have reported that LIPUS can reorganize the cytoskeleton and affect fibre distribution (Zhang et al., 2021). EphB4 is not only a membrane receptor but also a mechanoreceptor sensitive to stress through F-actin arrangement (Ohtani-Kaneko et al., 2017). EphrinB1 can change the distribution and morphology of F-actin, thus influencing proliferation (O'Neill et al., 2016). F-actin staining showed that LIPUS affected cytoskeletal structural through EphrinB2/EphB4. Hippo pathway is a mechanical stress sensing pathway closely related to the cytoskeleton; participates in the modulation of periodontal homeostasis (Deng et al., 2021). It has been reported that PDLCs sense and respond to mechanical forces during OTM with YAP (Huelter-Hassler et al., 2017). The TAZ modulates bone remodeling by activating Runx2 in OTM (Sun et al., 2018). Coincidentally, previous studies have shown that LIPUS can activate the Hippo pathway, thereby promoting the function of YAP/TAZ (Xu et al., 2018). Immunoblotting and immunofluorescence indicated that under the combination of EphrinB2-Fc, LIPUS reduced phospho-YAP in the cytoplasm and increased the accumulation of YAP in the nucleus via F-actin. At last, verteporfin, a pharmacological inhibitor of YAP, was used to confirm that the Hippo pathway is related to the osteogenic effect of LIPUS via EphrinB2/EphB4 signaling. This study observed the involvement of EphrinB2/EphB4 in BMSCs differentiation through F-actin and YAP, but this may be part of the regulatory mechanism of LIPUS on bone remodeling effect.

In conclusion, the results demonstrated that LIPUS upregulated EphB4 receptors on osteoblasts, which enhanced the EphB4-EphrinB2 interaction in the alveolar bone under orthodontic stress, leading to transduction of the LIPUS mechanical signals to the intracellular cytoskeleton and activation of the nuclear translocation of YAP in the Hippo pathway, thus regulating BMSCs migration and osteogenesis (Figure 7I). These results highlight the important role of EphrinB2/EphB4 signaling in LIPUS-induced modulation of bone homeostasis through osteoblast-osteoclast crosstalk and the potential value of LIPUS in orthodontic alveolar bone remodeling.

## 4 Materials and methods

### 4.1 Animals

Eight-week-old male Standard Deviation (SD) rats (weight 200 ± 20 g) for OTM model and C57BL/6 mice for primary cell isolation and culture were provided by the SJA Laboratory Animal CO., LTD (Hunan, China). All animals were housed under standard conditions: room temperature 22°C ± 2°C, 12 h light-dark cycle, water *ad libitum*, standard pellet diet. The experimental procedures were approved by the Ethics Committee of the College of Stomatology, Chongqing Medical University (Approval number: CQHS-REC-2022 (LSNo.104)). This study was carried out in compliance with the ARRIVE guidelines 2.0.

### 4.2 OTM

Significant intergroup differences of 0.6 mm (standard deviation = 0.1 mm) in control and 0.8 mm (standard deviation = 0.1 mm) in LIPUS were determined on the basis of published research (Arai et al., 2020). With an 80% power, an α of 0.05, and a 2-tailed test, 4 rats per group were recommended. After 1 week of adaptive feeding, 24 SD rats were anesthetised with 2% isoflurane gas inhalation and shallow grooves were carved in the gingival margin of the upper incisor and first molar. Ni-Ti closed coil springs (Shinye, Zhejiang, China) were fixed between the maxillary bilateral first molars and incisors. A force of 50 g was applied to induce mesial movement of the first molar. The orthodontic appliances were checked every day and were reinstalled or replaced in time if the appliances fell out or were damaged.

### 4.3 LIPUS treatment

The LIPUS generator was provided by the National Engineering Research Centre of Ultrasound Medicine, Chongqing Medical University (Chongqing, China). To reduce the impact of individual variation, each rat was exposed to LIPUS on the right side, and the left side was used as self-control with the LIPUS generator switched off (n = 24/group). LIPUS was performed from the first day after OTM. The ultrasonic transducer was placed in contact with the mucosa of the first molar through the couplant and LIPUS was applied at an intensity of 30 mW/cm^2^, frequency of 1.5 MHz, repetitive pulse frequency of 1.0 kHz and burst sine wave of 200 μs. The LIPUS stimulation was administrated for 30 min/d for 21 consecutive days. Three rats in each group were sacrificed with CO_2_ at appropriate time points (days 3, 7, 11, 15 and 21), and samples were obtained for histological studies.

### 4.4 Cell culture

For the culture of BMSCs, the femur was separated from male 8- to 10-week-old mice and bone marrow cells were flushed out with α-minimum essential medium (α-MEM) (C12571500BT, Gibco, Grand Island, NY, United States) supplemented with 1% penicillin/streptomycin and 10% fetal bovine serum (FBS) (BBP5, Moregate, New Zealand). The cell medium was changed every two-three days until the cells reached 80% confluence in approximately 7 days. The cells were seeded into plates at a ratio of 1:2 or 1:3. For osteoblast differentiation, BMSCs were cultured in osteogenic medium (α-MEM containing 10% FBS, 1% penicillin/streptomycin, 10 mM β-glycerophosphate, 50 mg/L ascorbic acid and 100 nM dexamethasone) for 7 days.

For BMMs culture, the femur was prepared as described above. The flushed bone marrow cells were cultured in a-MEM for 24 h and the floating cells were collected. The BMMs were obtained by gradient centrifugation with Histopaque (10771, Sigma, St. Louis, MO, United States), and were seeded into plates after resuspension. Osteoclast differentiation was achieved by culturing BMMs (2× 10^5^ per well in a 24-well plate) in osteoclastogenic medium (40 ng/mL M-CSF [CB34, Novoprotein, Suzhou, CN] + 100 ng/mL RANKL [CR06, Novoprotein, Suzhou, CN ]) for 7 days.

The method for building the osteoblast-osteoclast co-culture system has been widely described and analysed (Borciani et al., 2020; Hong et al., 2022). BMSC-derived osteoblasts were seeded at 2 × 10^4^ cells per well in a 24-well plate and BMMs were seeded on the attached osteoblasts (1 × 10^5^ cells per well) in osteoclastogenic medium (40 ng/mL M-CSF +100 ng/mL RANKL+ 10^−8^ mol/L 1,25(OH)-dihydroxyvitamin D3) on the following day.

### 4.5 LIPUS stimulation

The cells were irradiated with LIPUS (30 mW/cm^2^, 1.5 MHz) in a water bath, consistent with our previous research (Wang et al., 2018; Li et al., 2020; Ying et al., 2020). LIPUS stimulation was performed for 20 min daily for 7–14 days. Cells in the control group were treated with sham LIPUS stimulation (no energy output from the transducers).

### 4.6 EphrinB2-Fc administration

In order to stimulate forward signaling, soluble recombinant mouse ephrinB2-Fc chimera (496-EB, R&D, Minneapolis, MN, United States) was preclustered with His-Tag antibody (MAB050, R&D) and used at a final concentration of 1 μg/mL.

### 4.7 EphB4 shRNA transfection

Three shRNAs targeting EphB4 (NCBI gene ID: 13846) and non-targeted control shRNA were synthesized (Tsingke, Beijing, CN). The shRNA sequences were as follows:

#1: CCG​G-AGC​CAA​CAG​CCA​CTC​GAA​TAA-CTC​GAG-TTA​TTC​GAG​TGG​CTG​TTG​GCT-TTT​TTT;

#2: CCG​G-CCT​GTG​TCG​AAT​TGG​GTA​TTA-CTC​GAG-TAA​TAC​CCA​ATT​CGA​CAC​AGG-TTT​TTT;

#3: CCG​G-CGG​ATC​TGA​AAT​GGG​TGA​CTT-CTC​GAG-AAG​TCA​CCC​ATT​TCA​GAT​CCG-TTT​TTT.

BMSCs were seeded at a density of 2× 10^5^ in six-well plates and were transfected with pTSB -shEphB4 plasmid using Lipofectamine 3,000 (L3000015, Invitrogen, Carlsbad, CA, United States) within 16 h. For RNA and protein isolation, cells were harvested at 48 h and 72 h after transfection, respectively.

### 4.8 Statistical analyses

All quantitative data are presented as the mean ± SEM of at least three independent measurements. Differences between two groups were assessed with Student's t-tests and differences between four groups were assessed with one-way ANOVA. All statistical analyses were performed using Prism 8.0 software. Differences at *p* < 0.05 were considered statistically significant.

## Data Availability

The original contributions presented in the study are included in the article/Supplementary Material, further inquiries can be directed to the corresponding author.
