# Supplementary material for: Low-intensity pulsed ultrasound regulates osteoblast-osteoclast crosstalk via EphrinB2/EphB4 signaling for orthodontic alveolar bone remodeling
Source: Front Bioeng Biotechnol. 2023 Jun 23;11:1192720. doi: 10.3389/fbioe.2023.1192720 (PMC10326439; doi:10.3389/fbioe.2023.1192720)
Supplement: Supplementary file 1 [file DataSheet2.PDF]

Supplementary Material

Low-intensity Pulsed Ultrasound regulates osteoblast-osteoclast crosstalk via EphrinB2/EphB4 signaling for orthodontic alveolar bone remodeling

Jie Zhou[1, 2, 3], Yanlin Zhu[1, 2, 3], Dongqing Ai[1, 2, 3], Mengjiao Zhou[1, 2, 3], Han Li[1, 2, 3], Yiru Fu[1, 2, 3], Jinlin Song*

*** Correspondence:** Jinlin Song: songjinlin@hospital.cqmu.edu.cn

Figure S1. Uncropped blot of figure 3e

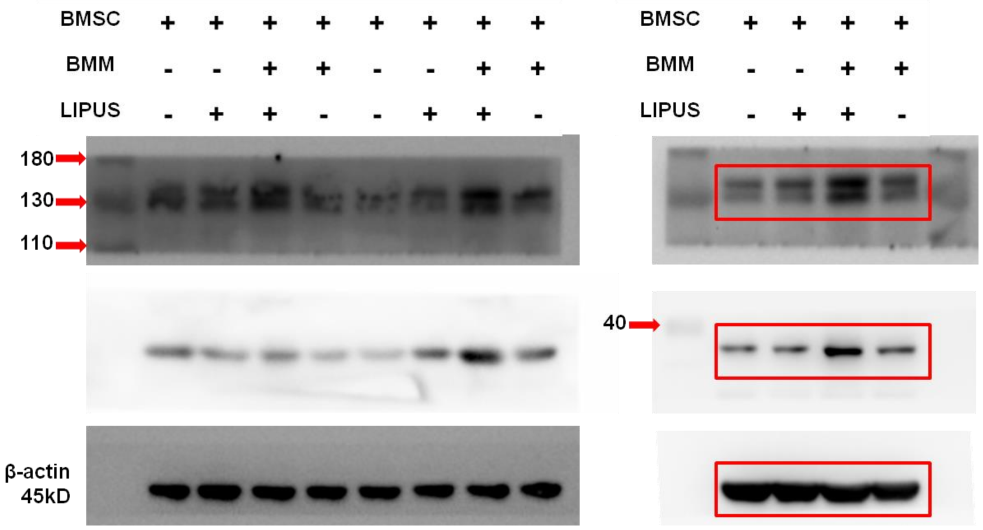

Figure S2. Uncropped blot of figure 5e

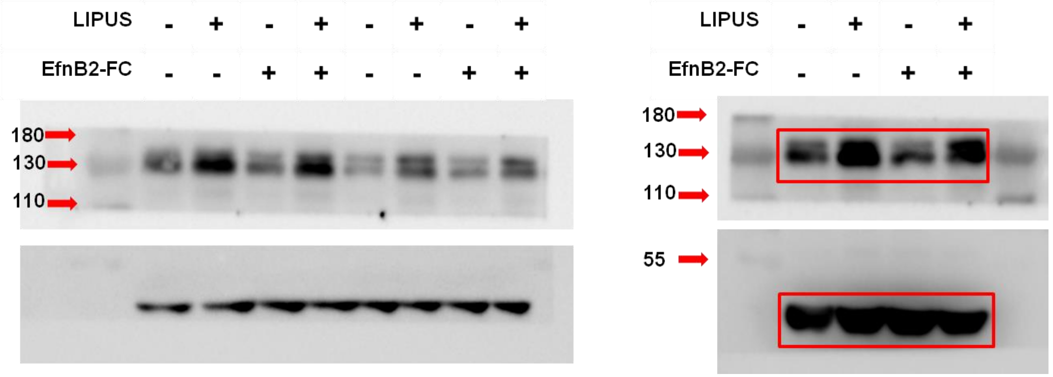

Figure S3. Uncropped blot of figure 6b

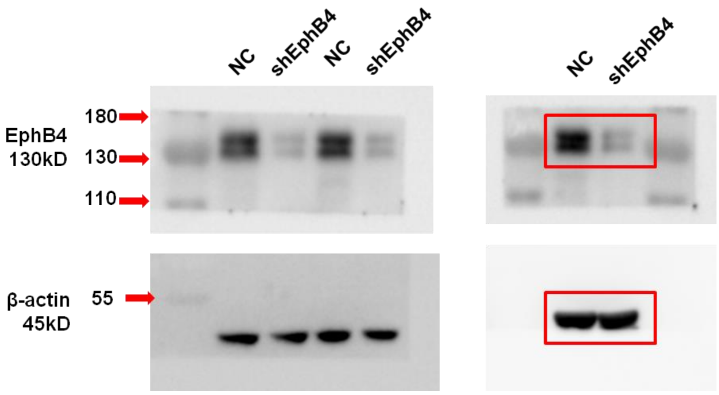

Figure S4. Uncropped blot of figure 7a

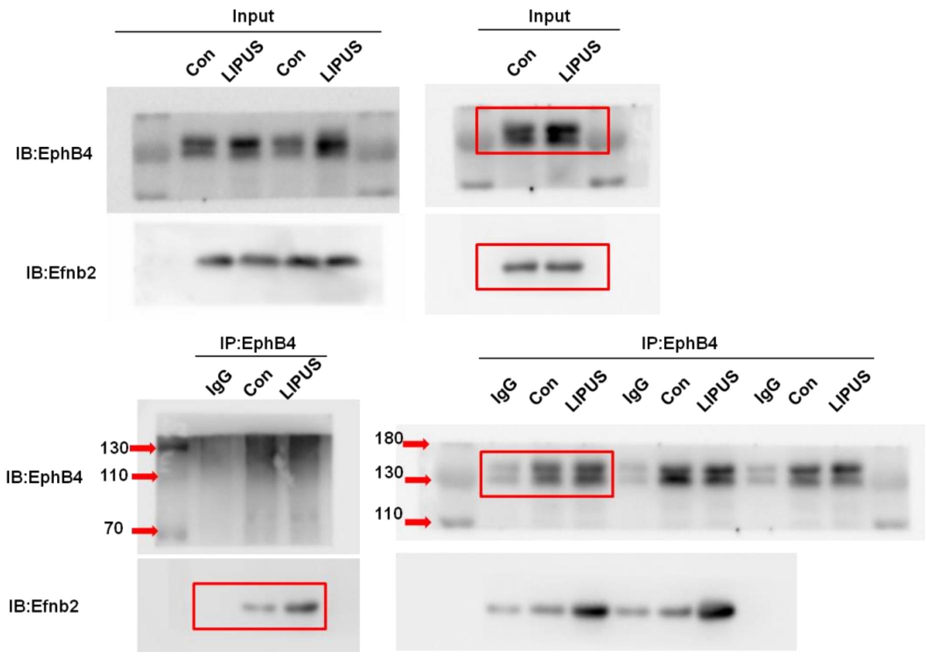

Figure S5. Uncropped blot of figure 7f

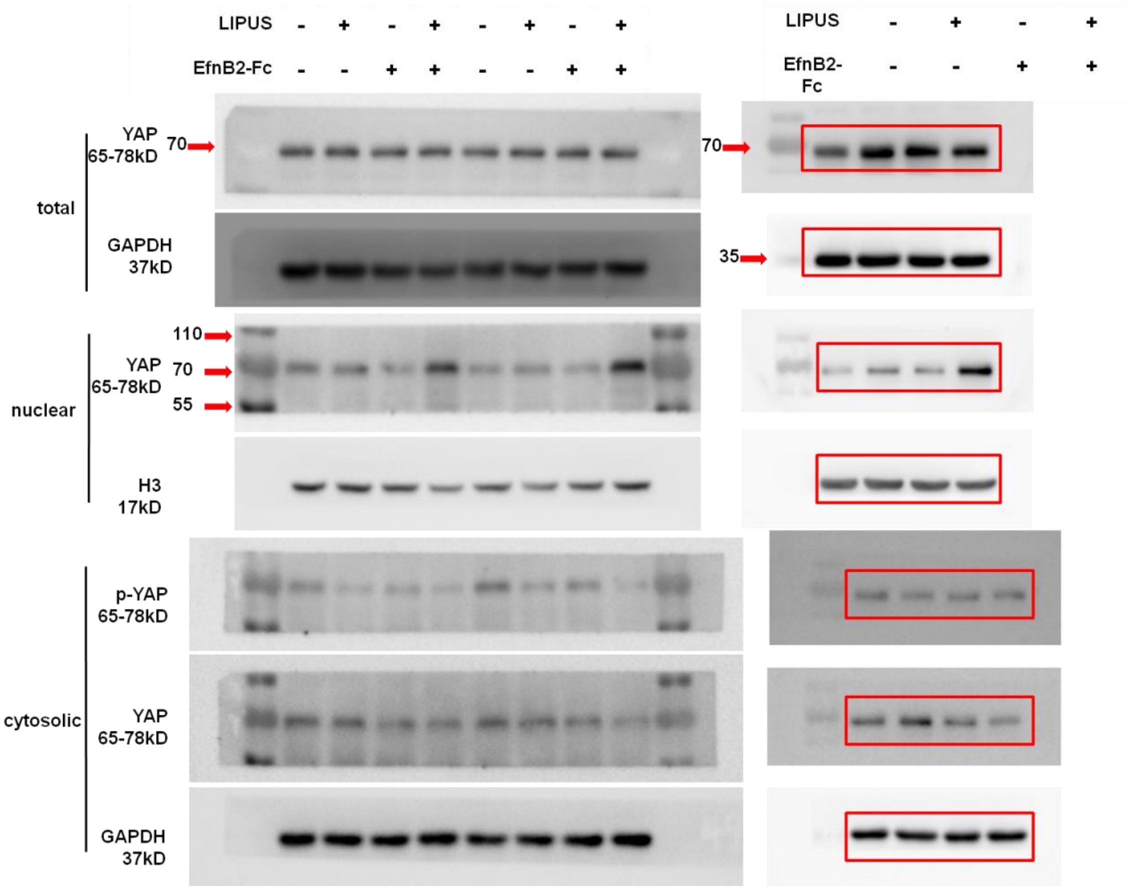

LIPUS
EfnB2-Fc
total
YAP 65-78kD
70
GAPDH 37kD
35
nuclear
110
YAP 65-78kD
70
55
H3 17kD
cytosolic
p-YAP 65-78kD
YAP 65-78kD
GAPDH 37kD
LIPUS
EfnB2-Fc
