# Supplementary material for: Low-intensity pulsed ultrasound regulates osteoblast-osteoclast crosstalk via EphrinB2/EphB4 signaling for orthodontic alveolar bone remodeling
Source: Front Bioeng Biotechnol. 2023 Jun 23;11:1192720. doi: 10.3389/fbioe.2023.1192720 (PMC10326439; doi:10.3389/fbioe.2023.1192720)
Supplement: Supplementary file 2 [file DataSheet1.PDF]

Supplementary Material

Low-intensity Pulsed Ultrasound regulates osteoblast-osteoclast crosstalk via EphrinB2/EphB4 signaling for orthodontic alveolar bone remodeling

Jie Zhou[1, 2, 3], Yanlin Zhu[1, 2, 3], Dongqing Ai[1, 2, 3], Mengjiao Zhou[1, 2, 3], Han Li[1, 2, 3], Yiru Fu[1, 2, 3], Jinlin Song*

*** Correspondence:** Jinlin Song: songjinlin@hospital.cqmu.edu.cn

1 Conventional materials and methods are described in detail:

1.1 Quantification of the tooth movement distance.

After the maxillary samples of each group were fixed and washed, they were photographed with a stereomicroscope (SteREO Discovery.V20, Zeiss, Germany) and analysed using microscopic measurement software (n = 3/group/time point). The distance from the distal contact point of the first molar to the proximal contact point of the second molar represented the range of OTM. The measurement was carried out by the same operator three times, and the average value was taken.

1.2 Micro-CT analysis

At the end of the animal experiment, CO_2 inhalation was used to sacrifice the rats (n = 6/group). After the orthodontic appliances were removed, the maxillae were trimmed to include the segment of the alveolar bone with three molars and the tissue 3 mm mesial to the first molar that contained the resorption and compensatory bone formation sites. The samples were fixed in 4% paraformaldehyde for 24 h and scanned using a micro-CT Scanner (vivaCT 40, SCANCO Medical AG, Switzerland). 3D reconstructions were generated by Amira software. A 200*200*400 μm volume set at a distance of 50 μm from the root furcation was defined as the region of interest (ROI) for analysis. BV/TV, Tb.Th., Tb.N. and Tb.Sp. of the alveolar bone were analysed, as previously described.[15,31]

1.3 Calcein and ARS labelling

Six rats were intraperitoneally injected with Calcein (20 mg/kg) (1461, Solarbio, Beijing, CN) one day before tooth movement, and ARS (25 mg/kg) (A5533, Sigma) was injected on the 13th day. The rats were sacrificed on the 15th day and the maxillae were cut in half along the sagittal plane. Then, the specimens were fixed, dehydrated and embedded in photopolymer resin. The specimens were sectioned at 5 μm thickness along the transversal direction with a hard tissue cutting and grinding system (E300CP/400CS, EXAKT, Germany) and were observed with a fluorescence microscope. The distance between the Calcein and ARS lines was taken as a measure of newly-formed bone.

1.4 Sample preparation and histology analysis

After micro-CT scanning, the samples were decalcified with ethylenediaminetetraacetate (EDTA; 10%, pH 7.4) for one month, embedded in paraffin and sectioned in the sagittal direction. Sections containing the mesial and distal buccal roots of the first molar, especially the 2-3 mm intact alveolar bone cortex of the mesial buccal roots, were selected. The sections were stained with HE and Masson for histologic observation, respectively. Images were acquired with a slide scanner (Slideview VS200, Olympus, Japan).

The paraffin sections were dewaxed, rehydrated, immersed in preheated buffer containing sodium citrate for antigen retrieval at 95℃ for 20 min, and blocked with hydrogen peroxide (3%). Then, the sections were incubated with mouse monoclonal IgG-anti EphB4 (1:200, sc-130081, Santa Cruz, CA, USA) or rabbit polyclonal IgG-anti ephrinB2 (1:200, D220598, Sango, Shanghai, CN) at 4℃ overnight, detected by biotinylated secondary antibody, and then visualized by a DAB Kit (ZLI-9017, ZSJQ-BIO, Beijing, CN), according to the manufacturer’s protocol. Finally, they were counter-stained with haematoxylin and Image J was employed to measure the mean optical density (OD) value and the size of the positive area.

1.5 ALP and ARS staining

ALP staining and measurement were performed after osteogenic induction for seven days. For activity measurement, cell lysates were obtained and measured by an Alkaline Phosphatase Assay Kit (A059-2-2, Jiancheng, Nanjing, CN). For ALP staining, the cells were washed with PBS, fixed with 4% paraformaldehyde for 10 min, and stained using a BCIP/NBT Alkaline Phosphatase Colour Development Kit (C3206, Beyotime, Shanghai, CN), according to the manufacturer's protocol. Calcium mineral deposition was determined after 21 days of osteogenic induction. The cells were washed with double distilled water, fixed with 4% paraformaldehyde for 15 min, and stained with 2% ARS (A5533, Sigma) for 30 min. The stained culture plates were imaged using scanner(V330, EPSON, Japan) and microscope (EVOS FL Auto, Thermo Fisher Scientific, USA), respectively.

1.6 TRAP staining

The differential level of osteoclasts was revealed by TRAP staining, which was performed using a Tartrate-Resistant Acid Phosphatase (TRAP) Stain Kit (G1492, Solarbio), according to the manufacturer’s instructions.

1.7 RT-qPCR analysis

For reverse transcription, 5 ug of total RNA in each group was submitted to a GoScript™ Reverse Transcription System (A5001, Promega, Madison, WI, USA). cDNA, as a template, was added into a mixture of SYBR green (A6001, Promega) and primer at a final concentration of 0.2 uM. RT-qPCR amplification was performed as follows: 94 ℃ for 20 s, 40 cycles of 10 s at 94 ℃ and 10 s at 55 ℃ and 10 s at 72 ℃, followed by the melting curve program. Relative expression levels were analysed by the ΔΔ-Ct method normalized to β-actin and the control group. Fold change data are presented as the mean ± SEM. The primer sequences used for the qPCR assays are shown in Table 1.

Table 1. Primer sequences.

Gene	Forward Primer sequences(5′-3′)	Reverse Primer sequences(5′-3′)

Gene	Forward Primer sequences(5′-3′)	Reverse Primer sequences(5′-3′)
EphB4	GTTCACCTTGCACTACCCCA	TCGGCAGCGTACAGCATAAG
EphrinB2	GCCAGACCAGACCAAGATGT	GCCCTCCAAAGACCCATTTG
Runx2	GGGAACCAAGAAGGCACAGA	GGATGAGGAATGCGCCCTAA
Col1a	CCCTGGTCCCTCTGGAAATG	GGACCTTTGCCCCCTTCTTT
Bglap	ACCTCACAGATGCCAAGCC	GCCGGAGTCTGTTCACTACC
Nfatc1	CCACTCCACCCACTTCTGAC	GTCGGGGAAAGAGACTTGGG
c-fos	CAGTCAAGAGCATCAGCAACG	CTCCCAGTCTGCTGCATAGAA
Ctsk	TGGAGGCGGCTATATGACCA	CCTTTGCCGTGGCGTTATAC
MMP-9	CTTCCCCAAAGACCTGAAAACC	CCATAGCGGTACAAGTATGCCT

1.8 Western blot analysis

At the end of the corresponding experiment, cells were lysed in RIPA buffer on ice, and total protein was extracted and quantified with a BCA Assay Kit (P0010, Beyotime). 20 ug of total protein in each sample was separated by SDS-PAGE and transferred to PVDF membranes. The samples were blocked by 5% BSA and incubated with primary antibody at 4℃ overnight. The antibodies used for Western blot analysis included mouse monoclonal anti-EphB4 (1:500, sc-130081, Santa Cruz, CA, USA), rabbit polyclonal anti-EphrinB2 (1:500, D220598, Sango, Shanghai, CN), anti-YAP (1:1000, 14074, CST, Danvers, MA, USA), anti-Phospho-YAP (Ser127) (1:1000, 13008, CST), anti-β-actin (1:1000, 8457, CST), anti-GAPDH (1:500, D110016, Sango) and anti-Histone H3 (1:1000, 4499, CST). Immunolabelling was detected using the ECL reagent and densitometry was performed by ImageJ.

1.9 Co-immunoprecipitation

The interaction between EphB4 and EphrinB2 was examined using the Co-IP protocol. The osteoblast-osteoclast co-culture system treated with LIPUS was washed with PBS and lysed with NP-40 (P0013F, Beyotime) on ice for 10 min. After centrifugation, the cellular extracts were collected, Pansorbin (507858, Sigma) was added, and mixing was performed at 4℃ for 1 h. The supernatant was obtained by centrifugation and was then incubated with anti-EphB4 antibody (1:50, sc-130081, Santa Cruz) and Protein A/G PLUS-Agarose (sc-2003, Santa Cruz) overnight at 4℃. Immune complexes were washed with ice-cold PBS three times, collected after centrifugation, resuspended with loading buffer, and boiled for 5 min for SDS-PAGE electrophoresis and immunoblotting analyses.

1.10 Wound healing and migration assay

The BMSC-derived osteoblastic cells with or without shEphb4 transfection were seeded into six-well plates. At 70% confluence, the medium was replaced to serum-free medium for 12 h of serum starvation. A straight-line was scratched in the well of each group by a ruler and 100 ul pipette tips. The wells were washed, and the floating debris was removed. Images were acquired by inverted microscope at 0 h as

control. The EphrinB2-Fc and LIPUS treatments were given according to the experimental design. The same scratch site was photographed again, and the scratch area was measured and calculated by ImageJ.

Cell migration was assessed using a Transwell assay. The BMSC-derived osteoblastic cells with or without shEphb4 transfection were seeded into inserts with a pore size of 8 μm (140629, Thermo, Waltham, MA, USA) within a 24-well Transwell culture chamber at $1*10^4$ cells per well. EphrinB2-Fc was added to the lower chamber and LIPUS treatment was implemented according to the study design. After incubation for 48 h, the non-migrated cells left on the inner side of the membrane at the bottom of the upper inserts were removed. The migrated cells were fixed with 4% paraformaldehyde for 15 min, stained with 0.5% crystal violet for 15 min, washed with PBS, and then observed and photographed using a microscope. Images were analysed using ImageJ software.

1.11 Immunofluorescence staining

The BMMs were cultured under the indicated conditions for seven days to examine F-Actin ring formation. After fixation with paraformaldehyde (4%) and permeabilization with Triton X-100 (0.1%), the osteoclasts from each group were stained with phalloidin-Alexa Fluor 555 (C2203, Beyotime) for F-actin and DAPI (C1005, Beyotime) for the nuclei. The F-Actin ring formation and nuclei were examined, and images were collected using a fluorescence microscope (EVOS FL Auto, Thermo Fisher Scientific, USA).

To explore the effect of LIPUS on the binding ability between the EphB4 receptor and EphrinB2 ligand, BMSC-derived osteoblastic cells treated with EphrinB2-Fc chimera protein cross-linking with His-Tag antibody and LIPUS were fixed with 4% paraformaldehyde and incubated with secondary antibody (NBP1-75181, Novus, Littleton, CO, USA) to detect the His-Tag antibody binding to the EphB4 receptor on the cell membrane.

The effects of LIPUS and the EphrinB2-Fc synergistic signals on the cytoskeleton distribution and nuclear location of YAP were observed by immunofluorescence. BMSC-derived osteoblastic cells from each group were fixed with paraformaldehyde (4%), permeabilized with Triton X-100 (0.5%), blocked with BSA (5%), incubated with rabbit monoclonal IgG-anti YAP (1:500, 14074, CST) at 4 ℃ overnight, and detected with Alexa Fluor 488-labelled goat anti-rabbit IgG secondary antibody (1:500, A0423, Beyotime) for 1 h at room temperature. The cytoskeletons were labelled with phalloidin-Alexa Fluor 555 for 20 min and the nuclei were stained with DAPI for 10 min. Imaging was performed using a fluorescence microscope.
